# A non-allelic homologous recombination-induced inversion in intron 22 of the *F8* gene is causative for haemophilia A in a Weimaraner male dog

**DOI:** 10.1371/journal.pone.0360975

**Published:** 2026-10-26

**Authors:** Sabrina Pach, Joel Fonseca Nogueira, Bertram Brenig

**Affiliations:** 1 Institute of Veterinary Medicine, Georg-August-University of Göttingen, Göttingen, Germany; 2 Universidade Federal do Vale do São-Francisco, Petrolina, Brazil; Chattogram Veterinary and Animal Sciences University, BANGLADESH

## Abstract

Haemophilia A is a severe coagulation disorder caused by partial or complete loss of function of the X-chromosomal *F8* gene. It is a relatively common coagulation disorder that has been reported in at least 17 dog breeds to date. However, only five distinct molecular causes have been identified so far. One molecular genetic cause is a large inversion, which has also been described in humans and was first studied in a research colony of Irish Setters at the University of North Carolina at Chapel Hill. However, the exact location of the inversion breakpoint junctions in dogs has not been determined. Here, we describe the molecular analysis of a case of haemophilia A in a Weimaraner dog family using long-read sequencing. We show that the cause of haemophilia A is an approximately 420kb inversion affecting the *F8* gene. The breakpoints of the inversion were identified in intron 22 at position 124,003,000 and between chloride intracellular channel 2 (*CLIC2*) and epsilon-trimethyllysine hydroxylase (*TMLHE*) at position 124,432,668 (NC_049260.1:g.124003000_124432668inv). As in humans, the inversion is most likely caused by non-allelic homologous recombination between intron 22 and a region approximately 420kb downstream of the *F8* gene. By identifying the inversion breakpoint junctions, it is now possible to test carriers of the disorder at the DNA level and thus prevent the risk of disease in male offspring.

## Introduction

Haemophilia A is an X-linked disorder [1] that affects the intrinsic coagulation pathway and is caused by a deficiency of coagulation factor VIII (F8). F8 is an essential cofactor (F8a) for the activation of F10 by F9a in the coagulation cascade [2]. Haemophilia A is the most common inherited bleeding disorder in humans and dogs [3–6]. The X-linked recessively inherited disorder has been described in humans, cats, dogs, horses, cattle, rats and sheep [7–22]. Clinical signs of haemophilia include persistent bleeding from the umbilical cord, prolonged bleeding time, variable lameness, haematoma formation, excessive bleeding after minor trauma or during second dentition, traumatic injury, venepuncture, and internal bleeding after trauma or surgery [23–25].

Coagulation factor VIII is produced by the *F8* gene, which is located on the long arm of the X-chromosome and is mainly synthesised in the liver and vascular endothelia. Due to its location on the X-chromosome, only males are affected, although in very rare cases heterozygous carriers can also develop a mild form of haemophilia A. Homozygous females can only be bred if an affected male is paired with a heterozygous female who are also referred to as conductors.

Factor VIII circulates in the blood bound to von Willebrand factor (vWF), which prevents its proteolysis and activation. In the event of injury, von Willebrand factor is cleaved off and factor VIII is activated. Activated factor VIII then acts as a cofactor, influencing the effect of activated factor IX on the activation of factor X, and is therefore an essential step in the blood coagulation cascade.

According to the CDC Haemophilia A Mutation Project (CHAMP), more than 4,000 causative variants in the *F8* gene had been described in humans by 2022 [6]. Single nucleotide variants in particular lead to haemophilia A in around 44% of cases. In a recent study single nucleotide variants account for approximately 26% of haemophilia A in humans [26]. In contrast to humans, the prevalence of haemophilia A in dogs is unknown. Apart from numerous case reports, only a few cases of haemophilia A in dogs have been characterised at the DNA level. Causative mutations for haemophilia A have been identified in Irish Setters, Mongrels, Redbone Coonhounds, Boxers, German Shepherds, Havanese, Wirehaired Dachshunds, Great Danes, Poodle mixes and Old English Shepherds, among others. These include two missense variants (German Shepherd, Boxer), two nonsense variants (German Shepherd, Bobtail), one insertion (Rhodesian Ridgeback), two deletions (Labrador Retriever, Border Collie) and one inversion (Irish Setter, Miniature Schnauzer) [27–37].

Haemophilia A has not yet been reported in Weimaraner dogs. In the present case, no causative variants in the coding region of the *F8* gene were detected by short-read sequencing, leaving an inversion in the *F8* gene as a possible cause [32]. In humans an intron 22 inversion (Inv22) accounts for at least approximately 40% of all haemophilia A cases [26,38,39]. Here we show that an inversion mediated by non-allelic homologous recombination (NAHR) between a GC-rich region in intron 22, which harbours the *F8A1* gene, and a homologous segment approximately 420kb distal to *F8* is causative for haemophilia A in the Weimaraner dog. While only a rough position of the inversion breakpoints has been determined in the experimental dog population in Chapel Hill using fibre FISH analysis, we were able to determine the exact inversion breakpoint junctions in the present study using long-read sequencing [32]. The inversion disrupts the *F8* gene. Although the inverted 5’ part (exons 1–22) of *F8* can still be expressed, the inversion results in a shortening of the FVIII C1 and A2 domains due to the absence of the coding regions of exons 23–26, leading to loss of function of the blood coagulation factor.

## Methods

### Ethical statement

The samples from the Weimaraner dogs were obtained *post mortem*. All other DNA samples were sourced from the institute's depository and stored as residual blood samples. The use of residual blood samples was approved by the Ethics Committee under no. E5-21 (2021-09-27). The study complies with German legal and ethical requirements for appropriate animal procedures.

### DNA samples

Samples from the deceased dogs were provided by the owners for analysis. We obtained samples of liver, spleen, kidney, muscle, and blood from the mother (Ne). A tooth, a soft toy, a chew toy, and a collar (with traces of hair) from the affected Weimaraner male dog (Ay) were available for DNA extraction.

### DNA extraction

DNA was extracted from all samples provided. Only severely degraded DNA could be extracted from the tissue samples (liver, kidney, spleen) of Ne, which was not suitable for further analysis. The blood sample had already coagulated and was therefore also unsuitable for DNA extraction. Sufficient DNA with a high molecular weight could be extracted from the muscle sample, which was used for DNA sequencing. Of the samples from Ay, only the tooth was suitable for DNA extraction. DNA extraction was performed using the Blood and Cell Culture DNA Midi Kit (Qiagen) with modifications according to a protocol from ONT. For DNA sequencing on the GridION (ONT), DNA was fragmented with a g-tube (Covaris) and then measured with the Qubit (Thermo Fisher Scientific) (Qubit 1x dsDNA Broad Range Kit). If necessary, the concentration was increased with VIVACON 500 (Sartorius) and then measured again with the Qubit. DNA was extracted from the tooth using the ChargeSwitch Forensic DNA Purification Kit (Invitrogen).

### Long-read DNA sequencing (Nanopore)

DNA sequencing was performed on a GridION (ONT). The Ligation Sequencing Kit V14 (ONT) was used for sequencing and performed according to the manufacturer's protocol. FlowCells FAY11344, FAY11779, FAY34763 and FAY34559 were used for sequencing the DNA of Ne and FlowCell FBA49383 was used for Ay. The settings for sequencing on the GridION were: run limit 72h, pore scan freq. 1.5h, minimum read length 1000 bp, read splitting ON, adaptive sampling ENRICH REFERENCE, basecalling HIGH-ACCURACY MODEL 400bps, Min Q score 9. The region NC_049260.1:g.123878920 to NC_049260.1:g.124943784 of the canine X chromosome (CFAX) was used as reference sequence (UU_Cfam_GSD_1.0, release 106, NC_049260.1). This region (1,064,864 bp) contains the centromeric part of the *GAB3* gene and ends downstream between the *VAMP7* and *IL9R* genes. The sequencing run was controlled and monitored with the GridION software MinKNOW (version 23.11.3, ONT). The number of reads per file was limited to 4000 and the fastq files obtained were merged into one file for further analysis.

### Bioinformatic analysis of the long-read sequencing data

The quality of the sequences (number of reads, size distribution, phred, etc.) was checked with the pycoQC software. The merged fastq files were assembled *de novo* with the Flye assembler (version 2.9.5) [40]. The resulting contigs were aligned to the selected region of the canine X chromosome using Sequencher (version 5.4.6, Gene Codes) and SeqMan NGen (version 18.0.3.2, DNASTAR). In addition, the contigs were merged into a local BLAST database and searched with the isolated exons of the *F8* gene and other genes of the CFAX region. The canine reference genome UU_Cfam_GSD_1.0/Dog (canFam4) (NC_049260.1) was used to align the sequence data with the online platform BLAT.

### PCR validation and Sanger sequencing of breakpoints

The primers cF8_contig26283_for (5’-CAGAGCCAGCGACTTCCC-3’) and cF8_contig26283_rev (5’-CAGAGAGACACCGGCCTC-3’) were used to validate the breakpoint of intron 22. The obtained PCR products were analysed with a Qsep Bio-Fragment Analyzer (BIOptic Inc.) using Q-Analyzer software (version 4.0.0/114). Amplicons were also analysed by Sanger sequencing and subsequent separation on an Applied Biosystems 3130xl DNA analyser. The sequences obtained were analysed using Sequencher software (version 5.4.6, Gene Codes) or SeqBuilder Pro (version 18.0.3.2, DNASTAR). Various dog DNAs of different breeds from the repository of the Veterinary Institute were used as positive and negative controls.

## Results

### DNA extraction and quality control

High-molecular-weight DNA is generally required for long-read sequencing. This depends, among other things, on the type of source material, its pre-analytical origin, and how it is obtained and stored. During DNA extraction from the samples available, sufficient quantities of high-molecular-weight DNA could be extracted from the muscle tissue sample of Ne (S1 Fig, lane 1). In contrast, the DNA isolates from the soft toy and tooth sample of Ay were already significantly degraded and showed a size distribution below 3kb (S1 Fig, lane 2, lane 3). An inhibitor control also showed that the soft toy DNA sample could not be amplified. No DNA could be extracted from the remaining samples of Ay (chew toy, collar with hair traces).

### Long-read sequencing of Ne

The sequencing libraries of purified and size-selected DNA from Ne were loaded onto four FlowCells. After the runs, the sequence data were first checked for quality using pycoQC (S2 Fig). The median fragment lengths obtained ranged from 1,135–1,548 bp and the Phred scores ranged from 13.76 to 16.56 (Table 1). The median fragment lengths were significantly shorter than the length of the selected DNA in all FlowCells, but all runs also contained larger proportions of reads with fragment lengths > 2,000 bp.

**Table 1:** Results of long-read sequence data of Ne using pycoQC.

FlowCell	Fragment length bp (mdn)	Phred score (mdn)
FAY11344	1,152	13.76
FAY11779	1,135	15.73
FAY34559	1,548	14.87
FAY34763	1,482	16.56

For further analysis, all filtered fastq files were merged into one dataset (Ne_all.fastq; FAY11344: 948,684 reads; FAY11779: 578,165 reads; FAY34763: 2,073,740 reads; FAY34559: 964,973 reads; total 4,565,562 reads; 21.63GB) and assembled *de novo* with flye. A total of 127,589 contigs with a total length of 1,013,464,080 bp (N50 = 10,464) and an average 5-fold coverage (mean coverage) were generated. The largest contig had a length of 161,742 bp.

For further analysis of the sequence data, a local BLAST database was created and searched with the 26 exons of the *F8* gene and selected exons of the *SMIM9*, *FUND2C*, *MTCP1, CMC4*, *BRCC3*, *RAB39B*, *CLIC2*, and *TMLHE* genes. These genes are located telomeric to the *F8* gene and should be located on the X-chromosome (CFAX) in the expected order. The aim of this analysis was to identify individual contigs in the dataset that could be assigned to the corresponding sections on CFAX. On the other hand, the Ne_all.fastq dataset was used to perform a direct alignment with the selected region on CFAX. This analysis should enable the assembly of a DNA region (scaffold) that is as complete and coherent as possible. S1 Table shows the contigs that could be assigned from the flye dataset to CFAX.

The isolated contigs covered approximately 47% of the selected region. For the *F8* gene, all exons except exons 5–14 and 20 could be assigned, and all telomeric genes except *VBP1* were represented. Another contig_5498 with a length of 16,847 bp was also identified, which contained exons 21 and 22 of the *F8* gene. This contig could not be optimally positioned because it contained an approximately 8 kb long part that appeared to have been incorrectly *de novo* assembled. Initially, no structural variants causative of haemophilia A could be identified from the available comparisons. However, it must be noted that Ne, as a female dog, carries two X chromosomes and would therefore be heterozygous for a structural variant (e.g., deletion, translocation, inversion, or similar). In addition, the chosen strategy mainly selected correctly assembled contigs that were comparable or identical to the reference genome.

Therefore, an alignment against the CFAX reference genome region was performed using the Ne_all.fastq and flye data sets. With this approach contig_26283 was identified (S2 Table). To verify that contig_26283 was indeed a continuous DNA sequence, the Ne_all.fastq dataset was aligned with contig_26283 as a reference sequence. As shown in S3 Fig, contig_26283 is generated by seven overlapping reads. Hence the sequences were assembled correctly by flye and the DNA fragment of contig_26283 is part of the genome of Ne. Contig_26283 was particularly interesting because the 5’ and 3’ regions belong to two separate genome segments in the *F8* region on CFAX, which are about 420kb apart (Fig 1). Fig 1 illustrates the orientations and locations of the breakpoint amplicon. The two opposing-coloured arrows in the centre indicate the inversion breakpoint. The left side of contig_26283 is homologous to the X-chromosomal region NC_049260.1:g.124,002,698 to NC_049260.1:g.124,003,000 in intron 22 of the *F8* gene, oriented towards the telomere. In contrast, the right-hand side of contig_26283 is homologous to a region spanning positions NC_049260.1:g.124,432,200 to NC_049260.1:g.124,432,668, approximately 420kb downstream, and in an inverted orientation, as indicated by the crossed lines. The homologous region is located between the genes for *CLIC2* and *TMLHE* (lower panel). For clarity, all other genes in the region, as well as the remaining parts of the *F8*, have been omitted. Note that the diagram is not to scale.

**Fig 1:**
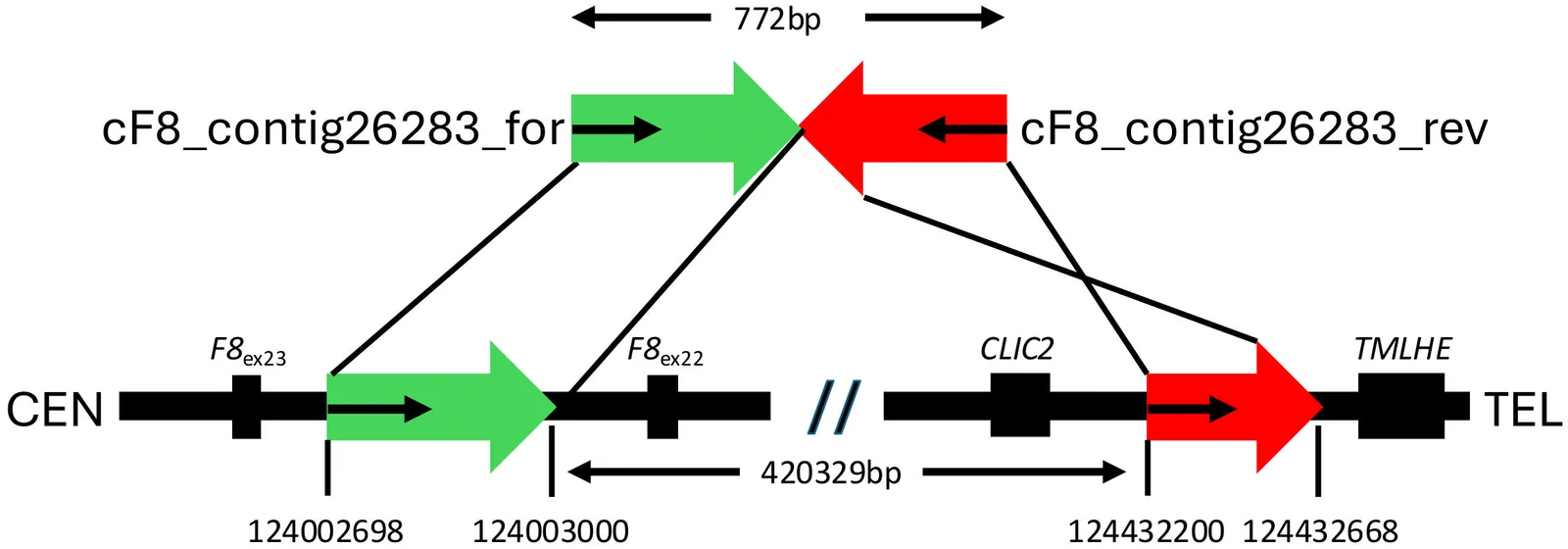
Graphical representation of the structure of contig_26283. The figure illustrates the composition of contig_26283. Contig_26283 can be placed in a chromosomal region of intron 22 of the *F8* gene and in a region between the *CLIC2* and *TMLHE* gene. For simplicity, the distances and sizes are not scaled. The positions refer to the canine reference genome (UU_Cfam_GSD_1.0, release 106, NC_049260.1).

Due to the significantly lower yield and quality of the DNA of Ay, only one FlowCell was loaded (FBA49383). The dataset Ay_all.fastq used for further analysis contained 1,545,829 reads (N50 = 342) with a median fragment length of 273 bp and a median Phred score = 14.295. Since the quality (especially fragment lengths) of the sequence data obtained was insufficient for assembly, the Ay_all.fastq dataset was gradually reduced to fragments of > 400, 500, 600, and 800 bp using Nanofilt software [41], and the number of resulting reads was determined. The remaining number of reads were 303,924 (>400 bp), 130,138 (>500 bp), 71,446 (>600 bp), and 33,202 (>800 bp). Since assembly only appeared to be useful for fragment lengths >800 bp and, due to the small number of sequences with this length, sufficient coverage of the chromosomal region was not to be expected and therefore the sequence data from Ay were not assembled. However, the isolated DNA was considered suitable for validation of potential structural variants by direct PCR.

### Validation of the inversion breakpoint using PCR and Sanger sequencing

To validate the inversion breakpoint in intron 22, primers cF8_contig26283_for and cF8_contig26283_rev were developed based on the sequence of contig_26283. It should be noted that identifying a suitable primer pair was not straightforward due to the extensive sequence homologies in the *F8* region. It proved impossible to develop primers that amplified only the region around the inversion breakpoint. The primers used had multiple binding sites within the region (S3 Table). Primer cF8_contig26283_for binds eight times (twice on the lower strand, six times on the upper strand), and primer cF8_contig26283_rev binds three times (once on the upper strand, twice on the lower strand) within the canine reference genome. However, most of the theoretically possible amplicons based on the primer positions would be too large, or the primer positions were too far apart and could therefore be disregarded. Nevertheless, on the wild-type chromosome six amplicons can be produced which, due to their size, can be generated in a standard PCR, including two amplicons (A2, A3) that were comparable in size to the inversion fragment (S4 Table). Sequence comparison of the potential amplicons (A2, A3) with the experimental amplicon of the inversion breakpoint revealed a clear sequence difference (S4 Fig). Alignment of the three amplicons showed that if the inversion is present, the amplicon contains a 22 bp repeat unit that does not occur in the potential amplicons of the reference sequence. Sequencing of the amplicon is therefore always mandatory to determine the presence of the inversion breakpoint.

S5 Fig illustrates the specific location of the inversion breakpoint amplicon using BLAT. The upper part of the figure (A) shows the DNA sequence with the positions of the primers used within contig_26283, generating a 772 bp amplicon. In panel (B) and (C) the alignments with the canine reference genome are shown. The 5´ part of the amplicon aligned to region NC_049260.1:g.124,002,698 to NC_049260.1:g.124,003,000 on CFAX whereas the reverse complement 3´ part aligned to region NC_049260.1:g.124,432,200 to NC_049260.1:g.124,432,668.

## Discussion

To date, only one case of haemophilia A in Weimaraner dogs has been reported, but the genetic cause remains unclear [42]. In the present case, short-read sequencing did not initially detect any variants in the coding regions of the *F8* gene. As an inversion in the *F8* gene caused by non-allelic homologous recombination has been described as a possible cause of haemophilia A in both humans and an experimental dog breeding population, we used long-read sequencing to identify such an event [32].

Detecting larger inversions is particularly challenging, as they do not usually result in deviating or extensive SNP variants [43,44]. Often, there are only minor sequence differences at the breakpoint junctions compared to the corresponding reference sequence [45,46]. In the present case, the situation was complicated by the fact that only the mother had sufficient DNA available for long-read sequencing and, consequently, heterozygosity for the inversion was to be expected [47]. In addition, there is an above-average number of repetitive regions and extensive sequence homologies, especially in the distal region of the X chromosome [48].

Although various bioinformatic approaches now exist for elucidating such structural variants, detection remains a non-trivial task [49]. Unfortunately, the use of standard programmes for aligning the *F8* region did not yield conclusive results [50]. We therefore initially assembled the long-reads *de novo* using flye [40]. To identify the breakpoint junctions, we created a local BLAST database with the generated contigs and searched it using non-repetitive DNA sequences from the potential breakpoint junctions, as well as exonic sequences of *F8* (exons 22–26), *CLIC2*, and *TMLHE*. The initial selection of DNA sequences for the BLAST search was based on the assumption that, as in humans, the inversion in the present case was most likely mediated by a GC-rich region in intron 22 of *F8*, where the *F8A1* gene and a homologous segment are located approximately 420kb distal to *F8* (Fig 2). In humans, the intron 22 inversion in the *F8* gene is caused by the homologous regions int22h1 in intron 22 and int22h2/3 between *CLIC2* and *TMLHE*. The int22h1 region (NC_000023.9:g.153,762,285–153,771,797) contains the *F8A1* gene. Int22h2/3 is located in the region NC_000023. 9:g.154,259,350–154,268,903 [51–53]. Both regions correspond to the chromosomal segments in dogs responsible for the inversion reported here. To identify additional parts of the *F8* gene and other genes of the X-chromosomal region within the contigs, the BLAST database was also searched using other DNA fragments from the canine reference sequence. Flye contigs with homologies to the selected DNA fragments were then aligned against the dog reference genome (UU_Cfam_GSD_1.0, release 106) using BLAT [54]. The chosen approach may be too complex in cases where the approximate location of the inversion event is unknown, but in the present case it led to a rapid reduction in the number of contigs to be analysed with the potential breakpoint junction.

**Fig 2:**
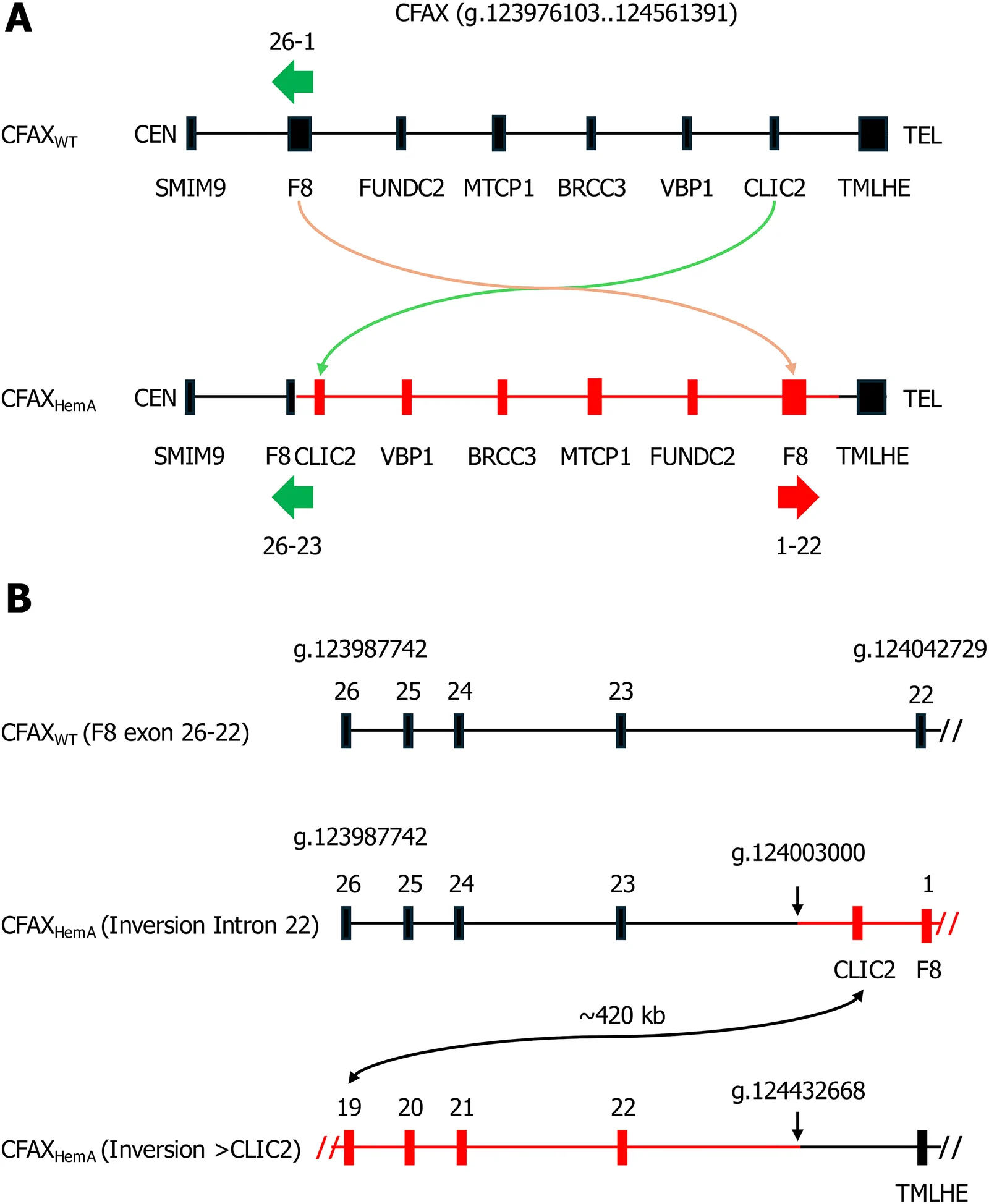
Graphical representation of chromosomal inversion in Ne and Ay. The figure illustrates the inversion process in Ne and Ay. A) CFAX_WT_ shows the natural arrangement (wild type, WT) of the genes on the canine X chromosome (CFAX) in the *F8* region. The *F8* gene contains 26 exons (1–26, green arrow) that are oriented from the telomere (TEL) toward the centromere (CEN). The inversion (CFAX_HemA_) causes a break in the *F8* gene and a region between the *CLIC2* and *TMLHE* genes. The inversion process is indicated by the curved arrows. The inversion rotates the *F8* gene in intron 22, so that exons 1–22 are oriented in the opposite direction. B) This part of the figure shows a detailed view of the inversion process with information on the approximate breakpoint positions. The positions refer to the dog reference genome (UU_Cfam_GSD_1.0, release 106, NC_049260.1).

In summary Ay was carrier of an inversion in intron 22 of the *F8* gene most likely caused by non-allelic homologous recombination and a homologous segment approximately 420kb distal from *F8*. The inversion disrupts the *F8* gene, resulting in a shortening of the FVIII C1 and A2 domains. The shortening of the C1 and A2 domains, encoded by exons 23–26, leads to loss of function of the coagulation factor. The inversion was inherited from its mother Ne, who was a carrier of the allele. Although it can be expected that the 5´ region of the *F8* gene (exons 1–22) might still be expressed, the inversion causes truncation of coagulation factor FVIII, resulting in loss of function and the development of haemophilia A. Although the occurrence of haemophilia A in Weimaraner dogs has been reported [42], the present findings show for the first time that the previously rarely described inversion in *F8* can also be the genetic cause of haemophilia A in this dog breed.

## Supporting information

S1 Fig Results of DNA extraction from the muscle sample of Ne as well as the tooth sample and soft toy of Ay. A) The muscle sample from the female dog Ne was extracted and purified as described above. In lane 1, 1µl of the DNA extract was separated. B) The DNA extracts from the soft toy (lane 2) and the tooth (lane 3) of Ay were applied directly after purification using a DNeasy column. All DNAs were separated on a 0.8% agarose gel (120V, 45 min). Using the size standard M (GeneRuler 1kb Plus DNA ladder, Thermo Fisher Scientific), the highest DNA quantity can be seen in the range between 5–20kb for Ne and 0.5–3kb for Ay. Raw gel images are shown in S6 Fig.
(TIF)

S2 Fig Quality control of output sequence data of Ne. Datasets of Ne were evaluated using pycoQC software. The figures show the distributions of fragment lengths and Phred scores for the four FlowCells used.
(TIF)

S3 Fig Sequence alignment of contig_26283. Contig_26283 was used to align the sequence data of the Ne_all.fastq dataset. The orientation of the individual sequences is indicated by green (forward) and red (reverse) arrows. The IDs of the reads are indicated.
(TIF)

S4 Fig Differences between amplicons resulting from the inversion breakpoint (Ne) and wild-type chromosomes (WT). The figure shows the DNA sequence of the amplicon generated by PCR primers cF8_contig26283_for and cF8_contig26283_rev (shown in bold and italics). The inversion breakpoint “G” is shown in bold and is within a 22 bp repeat (green, underlined). The six copies of the repeat are shown in green and cyan. In the wild-type chromosome, the amplicon lacks the repeat containing the breakpoint.
(TIF)

S5 Fig Alignment results using the 772 bp DNA sequence of the inversion breakpoint amplicon and BLAT [54]. (A) The DNA sequence shows the amplicon generated with primer cF8_contig26283_for and cF8_contig26283_rev (underlined). The nucleotide G shown in bold indicates the inversion breakpoint. (B) The 5´ amplicon fragment aligns from NC_049260.1:g.124,002,698 to NC_049260.1:g.124,003,000 (positive strand). (C) The 3´ amplicon fragment aligns from NC_049260.1:g.124,432,200 to NC_049260.1:g.124,432,668 (positive strand, reverse complement). Positions refer to canine reference genome UU_Cfam_GSD_1.0, CFAX, NC_049260.1.
(DOCX)

S6 Fig Raw gel images depicted in S1 Fig. (TIF)

S1 Table Contigs of the flye data set of Ne in the CFAX region 123,965,162–124,573,262. (DOCX)

S2 Table DNA-sequence of contig_26283. (DOCX)

S3 Table Binding sites of inversion breakpoint primers on CFAX. (XLSX)

S4 Table Potential amplicons resulting by using breakpoint primers. (XLSX)
