# Supplementary material for: A non-allelic homologous recombination-induced inversion in intron 22 of the F8 gene is causative for haemophilia A in a Weimaraner male dog
Source: PLoS One. 2026 Oct 26;21(10):e0360975. doi: 10.1371/journal.pone.0360975 (PMC13704881; doi:10.1371/journal.pone.0360975)
Supplement: Supplementary file 1 [file pone.0360975.s005.docx]

```
A)
cF8_contig26283_for
CAGAGCCAGCGACTTCCCGCCAGCGGCTTTTCTGGCGGGCGAGGGCCGGGCCGGGCCAATGGGGACGCGGTGACCTCATCAGGCCTGCGTGTCACCGCGTGCGCAGCGGGGCGGGGAGGGGGTGTGTCCCTCGGATACGGAGCGGGGCCTCCTCGCCCGCTGCTGAGGGCTCGGGTGCAGCCGGCCCAGCGCGCCGCACGGCGGTGCACCCCGGCGCTCCTCTCTGCGCCCCGACGCTGTTTCGGCAGCGCAGCCCGAGGCCCGTCTCTCTGCAGCCCGAGGTCGGACTCTCTGCAGCCCGAGGTCGGACTCTCTGCAGCCCGAGGTCGGACTCTCTGCAGCCCGAGGTCGGTCTCTCTGCAGCCCGAGGTCGGACTCTCTGCAGCCCGAGGTCGGACTCTCTGCAGCCCGAGGTCGGACTCTCTGTAGCCCGAGGTCGGTCTCTGCAGCCCGAGGTCGGACTCTCTGCAGCCCGAGGTCGGTCTCTGCAGCCCGAGGTCAGTCTCTCTACAGCCCGAGGTCGGTCTCTCTGCAGCGCGAGGCCCGTCTCTCCGCAGCCTGAGGTCGGTCTCTGCAGCCCGAGGTCGGACTTTCTGCAGCCCGAGGCCCGTCTCTCCGCAGCCCGAGGTCGGTCTCTGCAGCCCGAGGTCGGACACTCTGCAGCCCGAGTTCGGTCTCTCTGCAGCCCGAGGTCGGTCTCTCTGCAGCCTGAGGCCCGTCTGTCTGCAGCCCTTGCCCCGTCTCTCCGCAGCCCGAGGCCGGTGTCTCTCTG
     cF8_contig26283_rev

B)
000000001 cagagccagcgacttcccgccagcggcttttctggcgggcgagggccggg 000000050
>>>>>>>>> |||||||||||||||||||||||||||||||||||||||||||||||||| >>>>>>>>>
124002698 cagagccagcgacttcccgccagcggcttttctggcgggcgagggccggg 124002747

000000051 ccgggccaatggggacgcggtgacctcatcaggcctgcgtgtcaccgcgt 000000100
>>>>>>>>> |||||||||||||||||||||||||||||||||||||||||||||||||| >>>>>>>>>
124002748 ccgggccaatggggacgcggtgacctcatcaggcctgcgtgtcaccgcgt 124002797

000000101 gcgcagcggggcggggagggggtgtgtccctcggatacggagcggggcct 000000150
>>>>>>>>> |||||||||||||||||||||||||||||||||||||||||||||||||| >>>>>>>>>
124002798 gcgcagcggggcggggagggggtgtgtccctcggatacggagcggggcct 124002847

000000151 cctcgcccgctgctgagggctcgggtgcagccggcccagcgcgccgcacg 000000200
>>>>>>>>> |||||||||||||||||||||||||||||||||||||||||||||||||| >>>>>>>>>
124002848 cctcgcccgctgctgagggctcgggtgcagccggcccagcgcgccgcacg 124002897

000000201 gcggtgcaccccggcgctcctctctgcgccccgacgctgtttcggcagcg 000000250
>>>>>>>>> |||||||||||||||||||||||||||||||||||||||||||||||||| >>>>>>>>>
124002898 gcggtgcaccccggcgctcctctctgcgccccgacgctgtttcggcagcg 124002947

000000251 cagcccgaggcccgtctctctgcagcccgaggtcggactctctgcagccc 000000300
>>>>>>>>> |||||||||||||||||||||||||||||||||||||||||||||||||| >>>>>>>>>
124002948 cagcccgaggcccgtctctctgcagcccgaggtcggactctctgcagccc 124002997

000000301 gag 000000303
>>>>>>>>> ||| >>>>>>>>>
124002998 gag 124003000


C)
000000001 cagagagacaccggcctcgggctgcggagagacggggcaagggctgcaga 000000050
>>>>>>>>> |||||||||||||||||||||||||||||||||||||||||||||||||| >>>>>>>>>
124432200 cagagagacaccggcctcgggctgcggagagacggggcaagggctgcaga 124432249

000000051 cagacgggcctcaggctgcagagagaccgacctcgggctgcagagagacc 000000100
>>>>>>>>> |||||||||||||||||||||||||||||||||||||||||||||||||| >>>>>>>>>
124432250 cagacgggcctcaggctgcagagagaccgacctcgggctgcagagagacc 124432299

000000101 gaactcgggctgcagagtgtccgacctcgggctgcagagaccgacctcgg 000000150
>>>>>>>>> |||||||||||||||||||||||||||||||||||||||||||||||||| >>>>>>>>>
124432300 gaactcgggctgcagagtgtccgacctcgggctgcagagaccgacctcgg 124432349

000000151 gctgcggagagacgggcctcgggctgcagaaagtccgacctcgggctgca 000000200
>>>>>>>>> |||||||||||||||||||||||||||||||||||||||||||||||||| >>>>>>>>>
124432350 gctgcggagagacgggcctcgggctgcagaaagtccgacctcgggctgca 124432399

000000201 gagaccgacctcaggctgcggagagacgggcctcgcgctgcagagagacc 000000250
>>>>>>>>> |||||||||||||||||||||||||||||||||||||||||||||||||| >>>>>>>>>
124432400 gagaccgacctcaggctgcggagagacgggcctcgcgctgcagagagacc 124432449

000000251 gacctcgggctgtagagagactgacctcgggctgcagagaccgacctcgg 000000300
>>>>>>>>> |||||||||||||||||||||||||||||||||||||||||||||||||| >>>>>>>>>
124432450 gacctcgggctgtagagagactgacctcgggctgcagagaccgacctcgg 124432499

000000301 gctgcagagagtccgacctcgggctgcagagaccgacctcgggctacaga 000000350
>>>>>>>>> |||||||||||||||||||||||||||||||||||||||||||||||||| >>>>>>>>>
124432500 gctgcagagagtccgacctcgggctgcagagaccgacctcgggctacaga 124432549

000000351 gagtccgacctcgggctgcagagagtccgacctcgggctgcagagagtcc 000000400
>>>>>>>>> |||||||||||||||||||||||||||||||||||||||||||||||||| >>>>>>>>>
124432550 gagtccgacctcgggctgcagagagtccgacctcgggctgcagagagtcc 124432599

000000401 gacctcgggctgcagagagaccgacctcgggctgcagagagtccgacctc 000000450
>>>>>>>>> |||||||||||||||||||||||||||||||||||||||||||||||||| >>>>>>>>>
124432600 gacctcgggctgcagagagaccgacctcgggctgcagagagtccgacctc 124432649

000000451 gggctgcagagagtccgac 000000469
>>>>>>>>> ||||||||||||||||||| >>>>>>>>>
124432650 gggctgcagagagtccgac 124432668
```
