# Supplementary material for: A non-allelic homologous recombination-induced inversion in intron 22 of the F8 gene is causative for haemophilia A in a Weimaraner male dog
Source: PLoS One. 2026 Oct 26;21(10):e0360975. doi: 10.1371/journal.pone.0360975 (PMC13704881; doi:10.1371/journal.pone.0360975)
Supplement: Supplementary file 2 [file pone.0360975.s007.docx]

**S1 Table.** Contigs of the flye data set of Ne in the CFAX region 123,965,162 to 124,573,262

| contig (no.)^a)^ | Length (bp) | Position^b)^ | Gene/Exon^c)^ |
| --- | --- | --- | --- |
| 16549 | 18,415 | 123969893..123988307 | F8/26, SMIM9 |
| 126949 | 10,957 | 123989332..124000288 | F8/23-25 |
| 24681 | 9,104 | 124035737..124044840 | F8/22 |
| 24679 | 20,243 | 124049125..124069367 | F8/15-19 |
| 74992 | 15,981 | 124120881..124136861 | F8/3-4 |
| 133126 | 8,387 | 124137091..124145477 | F8/2 |
| 57078 | 58,135 | 124160835..124218969 | F8/1, FUNDC2, MTCP1/CMC4, BRCC3 |
| 13845 | 51,444 | 124219043..124270486 | BRCC3 |
| 13843 | 6,369 | 124272108..124278476 | intergenic [BRCC3, LOC102151175] |
| 3003 | 11,743 | 124283912..124295654 | LOC102151175 |
| 129501 | 47,849 | 124360228..124408076 | RAB39B, CLIC2 |
| 26263 | 4,412 | 124408076..124412487 | intergenic [CLIC2, LOC119878135] |
| 131097 | 3,402 | 124427547..124430948 | intergenic [LOC119878135 LOC119878040] |
| 38663 | 3,592 | 124430948..124434539 | LOC119878040 |
| 7905 | 17,612 | 124438814..124456425 | TMLHE/7-8 |
| Total | 287,645 |  |  |

a) Contig_5498 was not listed, since it could only be partially aligned (see text below). b) Positions refer to the dog reference genome UU_Cfam_GSD_1.0, Release 106, chromosome X (NC_049260.1). c) The listed genes are not always completely contained in the corresponding contig.
