# Supplementary material for: A non-allelic homologous recombination-induced inversion in intron 22 of the F8 gene is causative for haemophilia A in a Weimaraner male dog
Source: PLoS One. 2026 Oct 26;21(10):e0360975. doi: 10.1371/journal.pone.0360975 (PMC13704881; doi:10.1371/journal.pone.0360975)
Supplement: Supplementary file 3 [file pone.0360975.s008.docx]

**S2 Table.** DNA-sequence of contig_26283

| contig | Length (bp) | Sequence (5´-3´) |
| --- | --- | --- |
| 26283 | 4,109 | AACATTGCTGAAAGTCACTCGGGAGGCCCCGAATACGCGGGAAGCCCCGCCGCCCCCCCCCCCCCCCCCCGGTGTCAGGACCTGAACGTGTCCCATCGCCGAGTCGCGCCCAGGCGACAGACAGGCCGCACCCCCTCCGGGAAGCTCCCCCGCCCCGCCCTGCGCAGCCCGAGTGCAGGGGCCGCCTGGAGGGGCACCCAGAGGGAGGGCCCCGCCCGGTCCCGGCGCACCTGCAGCGCTCCCGCCGCGCCGACCCGCCGCTGCTGGCACCGCGGGGCGGCGCTCGGGACCCGGGGGCGCGACAGGCCCAGCCCGGGGGGGGGCGGGGGGGCGGGGGGGATCAGCTGGGCGGCCGCCTGCAGCCCGGGCTCGGGTCGGCAGCCCTGGGGGCGCGGGGGGAGCCCGCAGGCTCCACGGGGGCCCGGGTCAGCGGGCTGCAGCTGCCCGAGCTCCCTGTCCCTCGGGCCCCGCCCTGCCCCTCCCTACACACGGGGGACAGTGACCCCCAGGTGCCAGGTGCCGCGGCACAGCCGGCGCGGGGGCTCGCGAGGCCGCACCGGGAGGAGCAGGCGCGGGCCAGGGCCAGTGGGGCGGCGCGGCGGGAGCAGGGGGCCGGGTCAGCGCGCTGCCCAGACAGCGGGTGTCGCCCTGCCGTCGGGCCCCCCATCCCGCGCCCGGAGGGGCCCCGCGCGACCTTCGCCGCCGCCGCCGCCGCGCACGTCCGCCCGCAGGTGCCCCTCGCCGCCAGGGGGGGCGCGCAGTGGGGGCGAGTGCCCCTCGATGGACGTGCAGGGCGATCCCCGCTGCCTCGGGGGGCCTATTCCTGCTCCCTCGGGGGGACGGACCCCGACTCCTGTCCCTTGGCGGAGGGACCCCGACTCCTGTCCCTTGGGGGAGGGACCCTGACTCCTGTCCCTTGGGGGAGGGACCCCAACTCCTGATCCCTTGGTGGAGGGACCCCGATTCCTGATCCCTCGGGGGGAGGGACCCCGACTCCTGTCCCTTGGTGGAGGGACCCCGCTTCCTGATCGCTCGGGGGGAGGGACCCCAATTCCTGCTCCCTTGGGGGGGGGACCCGGGTTCCCGCTCCCTCGGAGAACAGCCCTCCTGAGAACACGGTGCCGAGGGAACGCGGCCGGCGCACACGGCCACCTACTGGTCCCAGGGTGGCCCCTGCAGCAGGTGTCCAGCGGACGCCAAGCGGCAGGGACAGGAGGCCGAGGAGCGGTCGGTCCCCGGGGGCAGGACGGGGTGCAGGGGGCAGTGAGCGCGCGGCGGCCGGGCCGTCTGTGCGGGGCGCTGCCGCGTGTGCAGCCGAGAGGGCCGACATGTCGGGGGTGCACGTGGGGGCGCGCGAGCTCCTGCACGCTCGTGGGTGGCGCCCCATGCCGTCCGCGCGGCGCAGAGCCAGCGACTTCCCGCCTTTTCTGGCGGGCGAGGGCCGGGCCAATGAGGACGCGGTGACCTCATCAGATCTGCGTGTCACCGCGTGCGCAGCGGGGAGGGGAGGGGGTGTCGCTTTTATACGGAGCGGGGCCTCCTCGCCCGCTGCTCAGAGCTCGGGAGCAGCCGGCCCAGCGCGCCGCACGGCGGTGCACCCCGGCGCTCCTCTCTGCGCCCCGACGCCGTCTTTCGGAAGCGCAGCCCGAGGTTGGTCTCTCCACAGCCCGAGGTTGGTCTCTCCGCAGCCCGAGGTCCGTCTTTCTGCAGCCCGAGGTCCGTCTTTTGCTCCCTCGGGCGGAGGGACCCCAATTCCCGCTCCCTTGGGGGTCCCGGATTCCTGCTCCCTCGAGGGGAGGGGCCCCGATTCCTGCTCCCGGGGGGGGGGGGGAAGAGGCCGTTATTCCTGCTCCCTGGGGGGGGGGTGCCCCGATTCCTGCTCCCTCAGGGGGAGGGGCCCGATTCCTGCTCTCTCGGGGGGAGGGACCCCGGTTCCCGCTCCCTCAGAGAACAGCCCTCCTGAGAACACGGTGCCGAGGGAACGCGGCCGGCGCACACGGCCACCTACTGGTCCCAGGGTGGCCCCTGCAGCAGGTGTCCAGCGGACGCCAAGCGGCAGGGACAGGAGGCCGAGGAGCGGTCGGTCCCCGGGGGCAGGACGGGGTGCAGGGGGCAGTGAGCGCGCGGCGGCCGGGCCGTCTGTGCGGGGCGGTGCCGCGTGTGCAGCCGAGAGGGCCGACATGTCGGGGGTGCACGTGGGGGCGCGCGAGCTCCTGCACGCTCGTCGGTGGCGCCCCATGCCGTCCGCGCGGCGCAGAGCCAGCGACTTCCCGCCAGCGGCTTTTCTGGCGGGCGAGGGCCGGGCCGGGCCAATGGGGACGCGGTGACCTCATCAGGCCTGCGTGTCACCGCGTGCGCAGCGGGGCGGGGAGGGGGTGTGTCCCTCGGATACGGAGCGGGGCCTCCTCGCCCGCTGCTGAGGGCTCGGGTGCAGCCGGCCCAGCGCGCCGCACGGCGGTGCACCCCGGCGCTCCTCTCTGCGCCCCGACGCTGTTTCGGCAGCGCAGCCCGAGGCCCGTCTCTCTGCAGCCCGAGGTCGGACTCTCTGCAGCCCGAGGTCGGACTCTCTGCAGCCCGAGGTCGGACTCTCTGCAGCCCGAGGTCGGTCTCTCTGCAGCCCGAGGTCGGACTCTCTGCAGCCCGAGGTCGGACTCTCTGCAGCCCGAGGTCGGACTCTCTGTAGCCCGAGGTCGGTCTCTGCAGCCCGAGGTCGGACTCTCTGCAGCCCGAGGTCGGTCTCTGCAGCCCGAGGTCAGTCTCTCTACAGCCCGAGGTCGGTCTCTCTGCAGCGCGAGGCCCGTCTCTCCGCAGCCTGAGGTCGGTCTCTGCAGCCCGAGGTCGGACTTTCTGCAGCCCGAGGCCCGTCTCTCCGCAGCCCGAGGTCGGTCTCTGCAGCCCGAGGTCGGACACTCTGCAGCCCGAGTTCGGTCTCTCTGCAGCCCGAGGTCGGTCTCTCTGCAGCCTGAGGCCCGTCTGTCTGCAGCCCTTGCCCCGTCTCTCCGCAGCCCGAGGCCGGTGTCTCTCTGTCCGCAGCCCGATGTCTCTGCAGCCCAAGCCCCATGTCCCCGCGGCCCGACGCCCGAGCGTCCGCAGCCCGGGGTCTCCGCAGCCCGAGGCCCGTGGCTCCGCGGCCCGACGCCCGAGCGTCCGCAGCCCGGGGTCTCCGCAGCCCGAGGCCCGTGGCTCCGCGGCCCGACGCCCGAGCGTCCGCAGCCCGGGGTCTCCGCAGCCCGAGGCCCGTGGCTCCGCGGCCCGACGCCCGAGCGTCCCCAGCCGGGCCGCAGCCGGGCCGCAGCCCGAGGCCCCGCGCCGAGCGCCGCCGAGGCCCCGCGCGCGGCTCAGGGGGAGCCGTGGAGGGCGGGCCGGCCGCCCGAGGTCGTGCCGCTCGCGCAGCGCCCGCGCGGGGCTGGCCTTCTCCGTGTGCCAGATGGAGCGCCTCCTGCGGGCCGGCCGCTACGCCAAGCGCCTGGGCGCGTCCGCCCCCATCTTCCTGGCGGCCGTCATCCAGTTCCTGACGGCCACCGTGCTGGGGCTGGCGGGCGACGAGGCCCGGCACAGGCGCACCGCGTACATCACCCCGGAGCTCGTGGACCGGGCGCTGCACAACCACGAGCTGCTCAGCGGCTTGTTCACGATGACCACCGTGTCCCAGGTGGCCCCGGCCCGGTACTAGCCGCCCCCGGCCCCTGATGCCCGCCGCCGCCCGGGGAGCCGCCGGGGGGGCTCGGTGACCCCCGCGTCCCCGCAATAAAAGGCGCAGAGACAACCAGACGTGTGCGCCTGGCTCACTGGGTGCGGGACGCGCCTCGGGGGGAGGGCGGGGGGGGGCGGGGGGGAGGTTGGGGTTTGGGGTTGTGGGCGAGGTTGGGGTGGGGGTGCGGGGCTCGGGGATGACGGGGGGTTGGGGTGCCCTCGGCGGTGCACGATGGGGGTGATGGAGGATTTGGGGTGTGGCGTGGGGCGCGGGTTCGTGGGTGTGGTGCGGGGTTCGGTCATGCGGTCCGGAGCATTGTTGGGGTGCAGGGACGGGGGCTGTGGCGGGGTCCGGCTGGGGTGCAGGGGTGGTGGTGGGGTCCGGCTGGGGTGCGGGGACGGGGACTGCTCGGGGT |
